# Indigenous Midwives and the Biomedical System among the Karamojong of Uganda: Introducing the Partnership Paradigm

**DOI:** 10.3389/fsoc.2021.670551

**Published:** 2021-06-18

**Authors:** Sally Graham, Robbie Davis-Floyd

**Affiliations:** ^1^ Department of Anthropology, University of London, London, United Kingdom; ^2^ Department of Anthropology, Rice University, Houston, TX, United States

**Keywords:** birth, TBA, traditional midwife, TBA trainings, Uganda, Karamoja, partnership paradigm

## Abstract

Certainly there can be no argument against every woman being attended at birth by a skilled birth attendant. Currently, as elsewhere, the Ugandan government favors a biomedical model of care to achieve this aim, even though the logistical realities in certain regions mitigate against its realisation. This article addresses the Indigenous midwives of the Karamojong tribe in Northeastern Uganda and their biosocial model of birth, and describes the need British midwife Sally Graham, who lived and worked with the Karamojong for many years, identified to facilitate “mutual accommodation” between biomedical staff and these midwives, who previously were reluctant to refer women to the hospital that serves their catchment area due to maltreatment by the biomedical practitioners there. This polarisation of service does not meet that society’s needs. We do not argue for the provision of a unilateral, top-down educational service, but rather for one that collaborates between the biosocial model of the Karamojong and the biomedical model supported by government legislation. We show that such a partnership is practical, safer, and harnesses the best and most economical and effective use of resources. In this article, we demonstrate the roles of the Indigenous midwives/traditional birth attendants (TBAs) and show that not only is marriage of the two systems both possible and desirable, but is also essential for meeting the needs of Karamojong women. The TBA is frequently all the skilled assistance available to these women, particularly during the rainy season when roads are impassable in rural South Karamoja. Without this skilled help, the incidence of maternal and infant mortality would undoubtedly increase. Ongoing training and supervision of the TBA/Indigenous midwife in best practices will ensure better care. We offer a way forward via the Partnership Paradigm (PP) that lead author Sally Graham designed in conjunction with the Indigenous midwives and biomedical staff with whom she worked, the development and characteristics of which this article describes.

## Introduction: The Impacts of Traditional Birth Attendant Training

Sally Graham and Robbie Davis-Floyd

Childbirth is a social as well as a physical event. Within it, tradition, culture, spiritual beliefs, and ritual all converge and are epitomised in the actions carried out by the traditional birth attendant (TBA)—better termed “traditional midwife” to acknowledge her role in her community as a midwife, or “Indigenous midwife” (IM) in the case of the Karamojong, as they are an Indigenous, semi-nomadic people. In this article, we will alternate between these two acronyms—TBA and IM—as international organizations such as WHO, USAID, and the International Confederation of Midwives (ICM) prefer to reserve the term “midwife” for those professionally trained in government-recognized institutions, and because the Indigenous midwives of Karamoja use the English term “traditional birth attendant” to refer to themselves.

In 1978, WHO stated:

Traditional medical practitioners and birth attendants are found in most societies. They are often part of the local community, culture and traditions, and continue to have a high social standing in many places, exerting considerable influence on local health practices … it is therefore well worthwhile exploring the possibilities of engaging them in primary health care and of training them accordingly. (ALMA-ATA 1978:47)

Thus, throughout the 1970s and 1980s, WHO encouraged TBA trainings, with the intention of effecting improvements in maternal and child health, even recommending expansion of the TBA’s role in other aspects of primary health care such as vaccination programmes and malaria prophylaxis (Greenwood et al., 1990). This type of initiative was taken up, for instance, in Nigeria, where traditional midwives were integrated into the organised health care sector with apparent success (Isenalumbe 1990). By 1972, 20 countries had trained traditional midwives and by 1994, 85% of low resource countries offered some type of TBA training. The main impetus for training was to reduce the high level of maternal mortality in such countries. In 1983, WHO estimated that 459 women per 100,000 live births died in childbirth in low resource countries as compared to 30/100,000 in high resource countries (Safe Motherhood Newsletter 1992).

Yet despite the training of thousands of TBAs, it was announced by WHO in 1994 that over 500,000 women still died from childbirth-related causes each year. By now—May 2021—this number has been reduced to around 239,000. This reduction has been attributed to the movement of birth into biomedical facilities, yet there are many other factors involved that we do not have space to address herein. WHO’s anticipated results of TBA trainings over a period of more than two decades had not materialized (Fleming 1994). The question is, Why? And the answer, as discussed below, lies in the culturally inappropriate, didactic, and biomedical orientation of these trainings that failed to take into account both the knowledge of the traditional midwives and their experiential modes of learning.

Even before the publication of that high mortality estimation, in their report on “Safe Motherhood,” WHO, UNICEF, and UNFPA produced a joint statement on TBA training that underlined their preferred Western model of care:

Because of the current shortage of professional midwives and institutional facilities to provide prenatal care and clean, safe deliveries as well as a variety of primary health care functions, WHO, UNICEF and UNFPA promote the training of traditional midwives in order to bridge the gap until there is access to acceptable, professional, modern health care services for all women and children. (*Safe Motherhood Newsletter* 1992:2)*.*

This statement makes clear that in the eyes of these organizations, the services of traditional midwives, though temporarily indispensable, were merely a stopgap until they could be replaced by professionals. Their skills were neither valued nor necessary in their own right and for their own sake. This attitude suggests a lack of understanding of the holism of the traditional midwives’ roles as culturally and socially specific to their context. This devaluation of traditional midwives has resulted in loss of their own self-confidence and by many of those who used to value and utilize their services (see also Ali et al., 2021; Ombere, 2021). Is this polarisation between the biomedical model and a biosocial one necessary? Why must there be an “either-or,” rather than a “both-and”? Why should there be this divisive approach at the point of care? In this article, we present a unifying solution, called by British-trained midwife Sally Graham, who developed this model in conjunction with the TBAs of Karamoja, Uganda, and the biomedical staff at the hospital that provided them with maternity care, the “Partnership Paradigm.”

The Partnership Paradigm (PP) is a model of mutual respect and accommodation between the formerly opposed hospital biomedical practitioners and the TBAs. As we describe below, it is composed of ongoing, trust-building dialogue between these two groups in which they learned about each other’s practices and techniques, resulting in the biomedical staff's appreciation for what the Karamojong TBAs already knew and the staff's ability to identify gaps in that knowledge and to fill them. It also resulted in the TBAs’ trusting of the biomedical staff and a great increase in their willingness to transport when needed, and in the staff's welcoming the TBAs to remain with their clients to provide continuity of care. Because this model is based on partnership, which includes the development of mutual accommodation and respect, the biomedical staff stopped blaming, shaming, and denigrating the TBAs as illiterate old women who knew nothing and who should not be attending births, and the TBAs came to see the biomedical staff as their allies and not their enemies.

Most of this article addresses Sally’s work with the Karamojong; Robbie Davis-Floyd’s contributions, based on her many years of both studying the literature on traditional midwives and working directly with many of them in Mexico (see e.g., Davis-Floyd, 2003b; Davis-Floyd, 2018), consist of adding in relevant theories and findings from that literature and from her fieldwork experiences, and extensive editing.

The current goals to address the still-too-high maternal mortality rate are directed toward contraception; prevention of unsafe terminations of pregnancy; an increase in the number of skilled birth attendants (SBAs), primarily professional midwives; better access to appropriate referral centres for complications; and most especially, a global drive toward facility birth and the elimination of the traditional or Indigenous midwives. Yet we are of the firm opinion that the key to positive change is embedded in the skills of the traditional midwives, despite the fact that international agencies concluded long ago that programmes for traditional midwives have not been effective in reducing mortality. Yet these training programmes, as many others have shown (see Jordan, 1993; Pigg, 1997; Cheyney et al., 2020) were taught by biomedical professionals who failed to appreciate the reality of the traditional midwives’ world. In so doing, they have de-skilled the already skilled local practitioners by changing practices that were in the main appropriate for the local situation. This is not to deny that some traditional midwifery practices are positively dangerous, such as putting dung on the umbilical cord to dry it in Karamoja, or putting a mixture of ash and cow urine in the mouth of a non-breathing newborn, as do some few traditional midwives in Pakistan (Ali et al., 2021). Yet a prescriptive approach to skill enhancement is counter-productive. Far from improving care, it has a detrimental impact because it may fracture the confidence and practice of the TBAs when they attempt to follow such programmes introduced from outside their own cultural and social reality. What they are taught is often, quite simply, irrelevant to them (Jordan, 1993; Lynch and Derveeuw, 1994; Cheyney et al., 2020).

The global development push that aims in the long term to replace the TBAs with professional, modern, biomedical health care services in itself poses questions about the quality and sustainability of biomedical services, which we further address below. Even in the long term, in many rural areas of the Global South, complete replacement is patently not feasible. For example, in Guatemala, 70% of births are still attended by traditional midwives (Daviss et al., 2021; in Kenya, the figure is 40% (Ombere, 2021), and in Pakistan, 24% (Ali et al., 2021)—all primarily in rural areas. Nevertheless, strenuous efforts are being directed toward the replacement of these practitioners. But replaced by whom? With biomedically trained personnel already in short supply even in the Global North, where are all these additional professionals coming from to replace the traditional midwives? Given that many biomedical birth facilities in rural areas are often short-staffed and under-supplied, and their practitioners often will not go into rural communities to provide care on site, this is indeed a pressing question.

Even when the intention is to offer referral facilities to the TBAs so that they can continue to practice and get the help they need, things commonly go badly wrong. We suggest that this is not only because referral facilities themselves are often inadequate and their personnel inadequately trained. It is also because the interface between the TBAs and those facilities has not grown out of dialogue and a genuine and mutual understanding between them, but rather from a cycle of misunderstanding of the real needs of the women and the TBAs who attend them.

### The Vicious Cycle of Failure

A vicious and self-perpetuating circle of failure is set into motion. Because they are often treated badly, shamed, and blamed by facility staff when they transfer, traditional midwives often seek biomedical help only as a last resort and because of this delay, the outcome of emergencies is poor or even worse, fatal (see Davis-Floyd, 2003b for examples). This reinforces the negative perceptions of the two parties and the whole dynamic between them becomes a self-fulfilling prophesy of failure. The problem is the chasm in understanding, appreciation, and mutual respect.

There is a solution to this problem. In this article, we argue that, despite major logistic problems such as transport difficulties, scarcity of medical resources, and political insecurity, a radical change in the perceptions of both biomedical practitioners and traditional midwives is possible. We base this assertion on evidence that will be adduced from Sally Graham’s reflections on her professional practice—a “*knowing in action”* (Schon, 1988)—and her work with the Karamojong TBAs/IMs and the biomedical practitioners—midwives and obstetricians—in South Karamoja, Uganda. This experience brought about a transformation in Sally’s own perceptions, enabling her to understand the two systems and to realise that no one system or model has all the answers, and so both must work together, as she describes in her own words below.

## The Karamoja of Uganda

Sally Graham, with contributions by Robbie Davis-Floyd

### The People and Their Environment

South Karamoja is a region of Northeastern Uganda covering an area of some 24,000 square kilometres and bordering on South Sudan and Kenya. To a Westerner, the environment appears hostile, and in fact life is precarious for both people and livestock. If the rains fail (and this happens every few years), the harvest will also fail, resulting in either a severe shortage of food or complete famine. Such an emergency then requires outside help for survival. This recurring need for assistance has deeply affected the people of Karamoja and encouraged from time to time a “beggar” mentality and undue dependency. This is possibly because of the ways in which aid has been given rather than the aid itself. It is difficult to know the reason, but this mindset still persists to some extent, even during the periods when conditions are reasonably favourable.

The Karamojong are pastoralists, which means that life centres on their cattle (Novelli, 1988; Graham, 1999) and they have a deep and personal love for their animals. Their economic survival is intrinsically linked with cattle. Cattle are the equivalent of “money in the bank” and an animal will be killed only as a last resort if all other means of subsistence have failed. In the normal course of events, only old or infirm cattle are slaughtered for food. Secondary subsistence is provided by small-scale agriculture, the staple cereal being sorghum. Women carry out most of the farming: they live in the settled areas, enjoy a rich communal life, and provide support for one another.

Cattle’s importance as a central feature of the culture influences every facet of life, both practically and emotionally. The number of animals owned represents a family’s wealth and prestige, and every effort is therefore made to increase the size of the herds, by whatever means. This pursuit of wealth results in frequent cattle raiding among tribal groups that undermines social stability in the region.

#### Social Organisation

The roles of women and men are radically different, yet complementary. Most women, elderly people and children live in groups and form a stable population in the settled areas in small villages, called *ngireria*. Their dwellings consist of circular huts constructed from wooden poles, mud, and thatch and fenced in by thorn bushes for security against surprise attacks from their enemies—the cattle raiders. The animals used for domestic purposes, such as milking cows, goats and chickens, live in the central part of this thorn-fenced area.

Men and their sons spend much of their time away from home in the cattle camps (*kraalsngawiyoi*) and graze their animals where there is available grass. They obtain water either from shallow wells that they dig or from bore holes. The younger women visit these men periodically, bringing provisions.

#### Status of Women

Women are highly valued by the society for two main reasons. The first is that daughters attract considerable wealth because cows are still paid as bride-price upon marriage. Secondly, there is a great respect for life among the Karamojong and childbirth is eagerly welcomed because children represent the future; they are the means of perpetuating themselves and ensuring their survival in old age when there is then a network of younger people to care for the elders.

## Methods and Materials

I went to Uganda in 1986 to work as a midwife in community health care in Karamoja and to conduct research for my PhD dissertation in anthropology (Graham, 1999). The Karamojong regions were part of its catchment area. Soon after my arrival, I began to hear about the TBAs. At first they were mysterious figures lurking in the background; I very much wanted to meet them but had no such luck until, six months after my arrival, a TBA brought a labouring woman with complications into the hospital. I was able to save the woman’s life, and thereafter the TBAs in the area began to seek my services and I was able to develop a trusting relationship with them. Soon I also developed an anthropological interest in their work and began to actively study it via participant observation and copious note-taking. I did manage to learn much of their language, but generally felt more able to communicate precisely when my Karamojong interpreter was present. My active fieldwork was conducted between 1988 and 1991, yet I have not felt comfortable with publishing it until now due to then-possible problems with the Ugandan government, which had outlawed TBA training by the time I was originally ready to publish. Yet such trainings continued in Karamoja for some years after this ban, and I did not wish to call attention to them at the time, as further described below.

Combined with other tools such as interviews and records, triangulation of data was possible. This eclectic approach enabled me to view the traditional midwives’ roles holistically in their full cultural context. It gave me a deeper understanding, rather than making my analysis purely on an evaluation of their practices. Important though these are, the Karamojong TBA's role is much more than an aggregate of practices. It is a way of life—one might even say the very reason for her existence.

The essence of the IM’s role can be understood only by living in her world. For this, adequate time is needed to understand that world and the reality of the circumstances in which she practices. Prolonged exposure is essential; otherwise understanding is both limited and distorted if research is confined to only part of the year. What is feasible in practical terms in the dry season may be totally untenable in the rainy season. This factor has relevance for anyone planning research or setting up a community-oriented programme. Seasonal change has a radical impact on life in Karamoja and totally alters the activities that can be undertaken. Ultimately, I lived with the Karamojong for more than two years during both types of seasons and so was aware of this factor. I did attend some births but tried to be as inconspicuous as possible, as I did not want to inhibit the two other midwives there. Fortunately, all births during my stay were normal, though I would possibly have been able to help with transport during the dry season if needed.

Extended exposure provided experience of the impact of ecological realities throughout the year. These included the paralysing of transport during the rainy season and the searing hot winds of the dry season, which cover everything with orange dust, make normal hygiene impossible, and problematize the process of walking several kilometres daily to collect sufficient water from the bore hole for survival.

To observe the women, pregnant or otherwise, in the planting season after the rains began and harvesting season when it was dry and crops were ready also helped me to explain the factors that affect childbirth and in consequence the IM’s part in it. In practical terms, accessibility to people—for meetings, social activities and medical services—are all affected by the season and the current priority for the population at that time. Thus, seasonal variations have an implication not only for research methodology but also for the planning, implementation and continuation of programmes. For example, it was not reasonable to try to organise meetings with the IMs for mutual knowledge exchange (described below) when all women were totally occupied planting crops—this was a crucial activity if they were to grow enough food to survive in the dry season. I was there long enough to be able to enter into the Karamojong’s cyclical time construct, develop ongoing dialogues with them, and participate in their activities. Thus I experienced and gathered information not only about the IMs and their work but also about their lives as women, especially in the family context.

### Representation

How we represented ourselves to each other had an impact on both myself and these midwives. For just as I was curious and interested in the IMs and how they practised, they too were interested in my world of midwifery—hence our ongoing dialogues that deepened their perspectives and mine. We both had our own versions of authoritative knowledge; according to Jordan (1993:154), that is “knowledge that within a community is considered legitimate, consequential, official, worthy of discussion and appropriate for justifying particular actions by people engaged in accomplishing the tasks at hand.”

My own experience as a British professional midwife enabled me to explore specific areas of interest critically, and because of my background of knowledge and expertise, I was in a position to compare biomedical approaches to the IMs’ care, and to evaluate what I was told and shown. The view of reality that the Indigenous midwives shared with me and the appropriateness of their methods and practices in turn gave me a clear understanding of why they did certain things in the geographical and cultural context of Karamoja. It soon became apparent to me that any attempt to impose a more technical model would have been not only totally inappropriate but also unethical.

For example, to prevent infection during birth, good hygiene is a basic aspect of physical care. In the Western context, this is easy to achieve because water and soap are usually readily available, labouring women can have a bath or shower, and the midwife can employ sterile techniques when assisting them. To expect the same preparations in a village hut is unrealistic and unrealisable when water has to be carried several kilometres and dust is everywhere. This does not mean that hygiene ceases to be an issue, but rather that the *principles* have to be shared, and not the techniques of the West, which are not sustainable in the Karamojong context. Eventually, after I had helped to establish full liaison with the hospital, the question of hygiene was partly solved by the provision of a freely given basic and renewable delivery pack containing soap, disposable sheeting, sterile disposable gloves, cotton wool swabs, new razor blades, and ligatures for tying the cord.

## The Acquisition and Application of the Indigenous Midwives’ Knowledge

The specific question of Indigenous knowledge and the importance of cultural reasons underlying childbirth practices has largely been neglected since the 1990s by development agencies in relation to traditional midwives. Instead, the extensive Annotated Bibliography by the World Health Organisation, 1985 gave a comprehensive account of how traditional midwives attend women and the practices they employ, without describing their cultural meanings and values. Pigg (1997:249) observed that in translating information about “local ideas and practices” into development discourse, “traditions” are systematically rendered as isolated “beliefs” and “customs.” Whilst sound practices are of course needed for safe care, what the TBA does must be understood within the context of her own social and ecological reality. The examination of practices alone is of limited value and therefore provides, at best, only a myopic understanding of the work of the TBAs. Unless there is better fundamental understanding of the TBAs’ world by planners and developers, biomedical trainings, in the countries where they still practice, will continue to serve as agents in the deconstruction of the TBAs’ societal orientations, and thereby will also continue to “alienate them in their own house” (Wigley, 1993:67).

The Karamojong TBAs are non-literate and uneducated in the formal sense; they are trained experientially in midwifery via apprenticeships with older, skilled IMs. In a Western culture, the interrelationship between practical and theoretical knowledge is of great importance in the context of professional midwifery education, in which theoretical and didactic aspects of learning commonly precede any practical experience (Jordan, 1993; Jordan, 1997). Among the Karamojong, as with most TBAs, their knowledge is acquired by *doing*, not by *talking about* doing (see Jordan, 1993; Cheyney et al., 2020 on the profound differences between didactic and experiential learning). And there is no dichotomy between ordinary everyday skills and those needed for birth attendance, as what the Karamojong learn from taking care of their animals translates easily into taking care of birthing women. This “doing” constitutes a highly developed art and skillset. This was demonstrated on numerous occasions when the TBAs carried out role play in response to a question, rather than simply explaining what they do, as seen in the following excerpt from my fieldnotes:

*The traditional midwives were asked to form five groups of six. Each group in turn was asked to explain what they do to help a woman give birth. Each group was provided with a home-made fetal doll, placenta and a canvas outline model of a woman and a uterus. There was a great deal of activity and arguing among the women. My translator listened in to the conversations and said that they were all talking about their own birthing experiences and births they had attended.*
*After some time, they got organised and began to role play the events surrounding birth. One woman knelt down and was supported by another from behind, while a third squatted in front making encouraging sounds. The woman supporting the “labouring” woman massaged her uterus from time to time and also joined in the encouragement. Eventually the fetal doll was produced from between the woman’s legs. There was no handling or assistance given to the baby for the actual birth, but once born it was handled with utmost care and tenderness. The “placenta” was dealt with quite differently. Immediate massage of the woman’s abdomen was carried out until the placenta was expelled.*

Whilst observing the IMs during such role play at meetings and workshops, it became clear to me that they were highly skilled and confident in the use of their hands. Such practices are distinct from the technical skills of biomedicine, which require manual dexterity, certainly, but not the intuitive movements needed by a traditional midwife. This dimension is of particular relevance, because the IM draws from the empirical knowledge gained via her own traditions and made specific by her own experiences. It is a knowing about how to cooperate with a bio-social event, rather than simply possessing a body of theoretical knowledge that has been specifically learnt but commonly out of the context of its eventual application. Within the TBAs’ practices, natural laws are observed, cooperated with and integrated into what I term *knowing action*. For example, all Karamojong men and women are involved in the care, bleeding, slaughter and utilisation of animals. Not only do they love and respect their animals, they also have an intimate and deep understanding of their physiology. Thus, much of the IMs’ knowledge is derived from their animal husbandry.

This understanding of mammalian physiology is manifest in the IMs’ ready understandings of the effects of maternal complications such as haemorrhage. For example, there was no need to explain the physiologic response of the body to bleeding, such as rapidity of the pulse, coldness of the skin, and eventual death if untreated. The IMs had both often witnessed this phenomenon and induced it when, for example, bleeding a goat or a cow to obtain its blood to mix with milk for their own diet. They also used this gentle means of bloodletting to slaughter a goat for a feast. Thus, clearly they were familiar with the effects of excessive bleeding in a postpartum woman, and had techniques for dealing with this, as I will describe below. These IMs are equipped with a literally “insider” knowledge of how the body functions. It was exciting to observe how their physiological knowledge gained from animal husbandry had been transferred and applied to the human situation.

### Visual Awareness in the Knowledge Process

I was curious to know if the IMs’ obvious powers of observation and visual awareness meant that they were also able to recognise and interpret pictures and models. So I designed and made some simple aids with which to test their perceptions. The following account, again from my fieldnotes, convincingly demonstrates that the IMs understand pictorial and other representations of subjects:

*Fifteen traditional midwives came for this meeting. We sat on the ground as usual in a circle and spent some time on courtesies and general chat. I told the woman that I had something to show them. This created excitement and curiosity. I had drawn a simple picture of a woman in local dress on a card (size A4). She wore many rows of colored beads and two items of detachable clothing, an apron and a wrap. The clothes were held onto the card by blue tac (a removable blue adhesive). Underneath the clothes was depicted a large pregnant uterus containing a curled up fetus. I held the card up and asked:*
*“What do you see?”*
*They reacted with intense interest, leaning forward, peering closely, some frowning, some smiling with obvious pleasure and commenting on the clothes and the beads. All agreed that what they saw was a woman.*
*I removed the clothes and asked:*
*“What do you see now?”*
*They said: “She has no umbilicus.”*
*This was followed by much concern until I explained that I had simply not drawn one in.*
*I asked, “Do you notice anything else?”*
*They became deeply concerned and several of them got up and touched parts of the picture where remnants of the blue tac had been left behind and exclaimed “neep, neep” (scabies).*
*They made no comment about the fact that the woman was pregnant, until I detached the fetus and they then nodded knowingly. I asked one of the group to put it back where it belonged and she immediately positioned it back in the uterus correctly. It was very significant that the group showed more interest in the “scabies” than in the depiction of a pregnant uterus, which suggests that the familiarity of a pregnancy could be dismissed as less of a problem than the possibility that the woman had a disease. (Village meeting with the traditional midwives, September 1989).*

This experience demonstrated not only their visual literacy, but also their great powers of observation. A Westerner would have ignored the blue tac, recognizing it as irrelevant, yet the traditional midwives, always observant, interpreted it as a skin disease. A Karamojong ignores nothing because survival may depend on the quality of observation. They quickly came to see the blue tac as irrelevant once I explained that this was the equivalent of, for example, mud being on the skin after working in their gardens (*manyattas*). Perhaps more important than visual literacy was their almost casual management of the model of the woman and fetus. This suggested that pregnancy is such an entirely normal event that it needed no special attention via the model.

Once I realised that the IMs understood and could interpret visual representations, I subsequently produced a more sophisticated life-sized model, which I used when discussing various midwifery complications.

*The same fifteen women again attended the meeting and as usual we sat on the ground in a circle, exchanging courtesies and local gossip while waiting for everyone to arrive. I was carrying a large plastic bag, which aroused immediate and intense curiosity. The traditional midwives had requested that we talk about postpartum haemorrhage. Since the previous meeting, we had made, out of canvas, a life-sized outline figure of a woman. It contained no details apart from showing the position of key landmarks for midwifery reference points such as the line of the breasts, the umbilicus and the pubis. In addition, there was a set of attachable organs consisting of a uterus, placenta, cord, and fetus which could be overlaid onto the figure and used for a wide range of demonstrations.*
*I did not show the figure to the group until the discussion was well under way, as I wanted to maintain a high level of curiosity. I asked them what happens to the body when a woman bleeds excessively after the birth. They know from practical experience of bleeding animals to death that there is a direct connection between blood loss and eventual exsanguination and death. I asked:*
*“What then do you do if the woman's life is failing because she is bleeding?”*
*They all said:*
*“We tie her limbs.”*
*At this point I decided to show them the life-sized figure, which was laid out on the ground in the middle of the circle. This caused great excitement. I asked if someone could show what they meant by “tying the limbs.” One of the group picked some grass and started to tie it around each of the limbs. She was joined by several others, who were murmuring approval. The figure was readily accepted as an aid for them to use in their role play.*

The IMs explained that they tied the limbs to ensure that there would be a greater blood supply going to the vital organs, thereby helping to counteract shock from blood loss. Uterine massage was combined with the administration of local herbs to make the uterus contract and stop bleeding. There was general approval by the group that these measures were effective. Certainly there is no argument with the principles of these practices; however, replacement of excessive blood loss requires more than just cessation of bleeding. It usually necessitates a blood transfusion if the woman is to survive, which the IM is powerless to provide.

Having established that the TBAs were able to recognise visual artifacts as educational tools, and involving them in role play to demonstrate elements of their practice and management of complications, made it possible to share our respective knowledge systems more readily.

### Authoritative Knowledge

Both Indigenous midwives and biomedical practitioners can be viewed as having authoritative knowledge, but the latter tend to practice standardized models of care that have little or no relationship with local cultures, beliefs, and traditions. In contrast, for the Karamojong IM, the authority of her knowledge lies in the fact that it is interactionally grounded in her cultural and environmental context (Jordan 1993). Dependence on the services of the IMs has particular importance in semi-nomadic pastoralist cultures such as the Karamojong. Spooner (1973) and others have pointed out that nomadism has a sound ecological basis, and that the health of nomads is closely connected with the health of their environment. MacDonald (1994) observed that local needs should be at the heart of the health services, and local perceptions, wisdom and experiences should be valued. This concept is certainly reflected in the ways in which these TBAs, referred to locally as *Akimat* in the singular and *Ngakimat* in the plural (which translates into English as “old woman”) function in the specific context of Karamoja. Their role is akin to parenting, not only in the biological sense of bringing forth a child, but also in the wider context of nurturing women and their babies in the physical and cultural milieu of the reproductive period of their lives. The IM is an intrinsic player in all such activities, because she practices in her capacities both as a woman and as a midwife. In this comprehensive role, she is one of the cornerstones of stability in Karamojong society. The importance of the IM as a major contributor and component of health care programmes and her more fundamental role as a cultural agent within her own community (Laderman 1983; Benoit 1989; Vincent-Priya 1991; Cominsky 2016) must always be taken into account.

The linking of direct cause and effect might imply that there are no other factors involved in mortality apart from the IMs’ behavior. However, Kwast (1991) showed that the IMs’ performance cannot be blamed when other crucial factors such as accessible referral facilities are not available. Yet crucial though backup and transport are, they are still only effective if they are efficient. Poor services, which are unfortunately all too common, provide a false sense of security. Biomedical facilities and transport systems must be of good quality before being valued and utilized by local people. If they are, then rather than replacing Indigenous systems, biomedical systems can be integrated into local systems that already work well. I was acutely aware of this factor, and recognised that it might be possible to harness the potential inherent in the Karamojong situation, given the excellence of the biomedical facility available to them, at least during the dry season.

## Indigenous Midwifery Systems

Indigenous systems can and do work, because the people involved are the only ones truly in a position to perceive with any accuracy the potential of their own situation and what is possible, or more importantly, not possible. There has to be a match between their view of reality and programmers’ versions of reality. Systems must not be based on the program developer’s medically ethnocentric view. It must be clearly acknowledged that people who have lived in an area for generations have knowledge that counts and should not be discounted. Until this happens, it is quite likely that little further progress in reducing maternal mortality will be made, as most maternal deaths occur in sub-Saharan African countries where IMs still attend large numbers of births in rural areas, with little or no access to *efficient* biomedical facilities. Thus it is clear that IMs in such countries, including Uganda, must be trained in ways that first take into account what they already know, and work from there, rather than imposing top-down biomedical information in ways that fail to take traditional knowledge systems into account. The key to understanding and achieving this sort of “mutual accommodation” (Jordan 1993) is quite simply dialogue.

### Problems With “Marrying” Indigenous and Biomedical Systems

It is ideal to promote and foster Indigenous systems of care in order to be culturally sensitive and safe, and ecologically realistic. The fact remains, however, that to rely on these unilaterally is not the solution either. To disregard the advances in biomedicine and the advantages that biomedical assistance can provide would be as unethical, detrimental, and ineffective as attempting to implement a biomedical model of care in remote rural regions. In both scenarios, one falls into the same trap of polarisation: the either/or approach. However, the major problems of marrying the two systems for the benefit of both are transport difficulties (or even total absence of transport) and disappointment with the referral services that reluctant TBAs have been persuaded to use. This, again, is because too often the TBA is met with rudeness and disrespect by personnel at referral centres, and/or the referral centre itself is ill-equipped both in needed supplies and in expertise. These deficiencies ensure that the TBA retreats from what she perceives as poor care and fails to use the system. The bottom line is that the motivation for cooperation between the TBAs and the biomedical system is not generated when there is no mutuality or accommodation (Jordan 1993) between the two players.

This factor was one of the main challenges I faced when attempting to initiate a referral system that worked both for the Karamojong and for the biomedical personnel in the referral hospital. This challenge was overcome by the extensive dialogues that I worked to facilitate over a period of 18 months. *There is no shortcut to the development of trust.* It demands a considerable investment of time and attention, which fortunately I recognised and was able to provide. Instead of a polarised approach, my solution in Karamoja was the development of what I called the Partnership Paradigm and its implementation, which brought about an ideal type of mutual accommodation between the TBAs and the biomedical staff of their referral hospital. In what follows, I use my experience of living and working among the IMs of Karamoja to demonstrate how this can be so.

## Ongoing Meetings

My work with the IMs settled into a pattern of weekly meetings, usually during the early morning hours because as the sun found its way above the horizon, it became very hot and work was just not possible. I tried where possible to use their recent experiences as a basis for discussion, and on the whole this worked very well. It quickly became evident that they were genuinely interested in finding new ways of dealing with problems and in sharing the knowledge they already had. Our role play was not only informative but fun, and revealed the beauty of their culture; for example, once a pregnancy becomes public, the pregnant woman is adorned with a leafy garland and given a celebratory blessing by an IM. This ritual, though not specifically religious, carries deep spiritual significance and solemnity, and constitutes a public recognition of a major life event.

On occasion, our meetings were slow to get going because the IMs were sleepy from their breakfast of local beer, and so to stimulate them, on impulse I stood up and said, “Join me in one of my dances!” They all stood up excitedly, and I then proceeded to demonstrate the “Hokey Kokey”—a boisterous dance that involves much movement and jumping up and down. After only a few minutes, the whole group entered into it with enthusiasm. Thereafter, it became our signature song and dance, and whenever we met they wanted to begin with it. Even years later, when I was simply visiting them, they would laugh and start singing the tune to the dance. Yet on the first occasion, after they all collapsed laughing, one of them said, “You taught us your dance—you must now learn ours!” This unlocked a whole celebratory aspect of their culture, as they performed one elaborate dance after another, most of which depicted the movements of cows. My joining in and dancing with them signalled another step in the mutual acceptance of our respective cultures as well as in the midwifery sphere. One of the constant features of their dancing is that sometimes they rise as much as a foot or more off the ground—even the very elderly. When I tried to join in, after only a few minutes I had to sit down, exhausted, and my “weakness” thus became the subject of much merriment and teasing.

I learned over time that the Karamojong have little understanding of germs or the concept of infection. Cow dung is precious and has many uses—for example, to anoint a person when wet, to light a fire when dried, to form part of hut-building, and, to my dismay, for putting on the umbilical cord. I could not help but wonder how so many babies escaped without infection—I could only imagine that their immunity was high. Another problem was that the cord was cut using an unsterile sharp instrument that was also used for other purposes. The cord of a male child was cut with a spear, whereas that of a female was cut with a knife. Yet despite the cultural meanings of such practices, neonatal tetanus sometimes occurred and with it a high mortality.

Clearly these practices needed changing, but the process was not simple. For a start, as previously noted, water was very scarce and had to be carried several kilometres from the bore hole. Even when it was available, there was no way to sterlise it because to do so meant lighting a fire, and fuel was both expensive and hard to come by. Fire was used for making the local beer—a major ingredient of the Karamojong diet. To them, this was a far more important use than washing, which was alien to them—their skin was a stranger to water except when it rained. Instead, oil was used to smear their whole body to stop the drying effect of the constant hot wind. I did discover that they liked soap, which they used for the occasional washing of clothes, which were then laid out in the sun to dry. Later, more bore holes were drilled and water became more accessible. Because I had some monetary resources to draw on (and before the previously mentioned provision of the birth packs), I decided as a goodwill gesture to provide a small gift for each TBA on a monthly basis. I gave each of them a small bar of soap, a packet of much-sought-after salt, and a packet of snuff, which they very much enjoyed using.

I had to figure out a way to introduce the alien question of hygiene, and, as often happened, an incident arose that gave credibility to my efforts. One day toward the end of a meeting, two women appeared. One of them was obviously very ill and sank down at the edge of the group. She had given birth a few days before and had developed a high fever. She had visited the local shaman, who told her that she was suffering from the evil eye, and to remove this curse, he had made a series of cuts across her abdomen and genitalia. This made her fever much worse. As a last resort, she decided to come to me for help. It was obvious to me that a course of antibiotics was needed to treat both the fever and the ill effects of the cuts made by the witch doctor. I was able to both treat the woman and to use what had happened as an example of a better alternative to the treatment she had received. I laid out some antibiotic tablets and explained to the IMs that these would make the woman better in a few days, and asked two of them to check up on her progress and report back. I also asked which of the two treatments they thought was best—to be cut in lots of places or to swallow some tablets. The group became very thoughtful, mulling over what I had asked. Tribal peoples are inherently conservative. They will only change if they are convinced that it is to a better way. To my profound relief, a week later when I met the group again, the same woman had come along to see me. She had fully recovered and became a strong witness to the efficacy of appropriately used biomedical technology. This experiential incident was more powerful than any amount of discussion, and noticeably sharpened the women’s interest in what I had to offer.

## Roles and Practices: Traditional Midwives as Conduits for Cultural Continuity

It is not always possible to elicit a reason for many of the traditional midwives’ actions. Often, when enquiring why a certain ritual is performed, the reply is simply, “It is done like this.” Even so, it is possible to get an insight into the reasons as one becomes more familiar with the culture. For example, once the cord is cut and the baby is put to the breast, names are whispered in the baby’s ear and when the newborn begins to suck, this signals acceptance of a particular name. This invitation to the baby to choose its own name could be seen to symbolise the egalitarian nature of Karamojong society, in which dialogue—even with a newborn—plays a central and crucial role.

In the performance of her midwifery work, the TBA is a conduit for the rite of passage of birth and the transmission of rituals and customs to the next generation. Davis-Floyd (2003a) and Cheyney (2011) describe childbirth as a means of enculturation. They present the notion that whoever has access to the control of birth also has a powerful tool for the reaffirmation or reconstruction of a society and its culture. Robbie demonstrates the power of technocratic biomedicine in this task, while Melissa Cheyney shows how the rituals of home birth can work to construct and enact a holistic, family-centred model of both birth and life. Thus the IM exercises a powerful influence for ensuring preservation and continuity of culture within her society. She does this by example and by teaching selected younger women her skills. For instance, when I returned to Karamoja after an absence of several years, some of the elder traditional midwives came to meet me with their adult daughters, who, they proudly told me, were assisting them with births and would one day take over from them.

This cultural continuity is being challenged as social change permeates the Karamojong and other rural societies. The dual role of the IM as both midwife and woman is then also crucial, as it is vital for the benefit of rural communities to have such “birth models that work” (Davis-Floyd et al., 2009), specifically because they are culturally appropriate and can be passed onto the next generation. Yet in many traditional or Indigenous societies, the IMs who have been phased out of practice are simply dying without passing on their knowledge and skills to anyone. Thus entire bodies of knowledge about birth that may have contemporary relevance are being lost.

I re-emphasize here that the strength of the Indigenous models of midwifery is that they evolved and were acquired in their cultural context, and so are appropriate and in harmony with the values of the society. Nonetheless, maternal and infant deaths certainly occur, revealing limitations and shortcomings in the Indigenous knowledge system, just as there are faults inherent in the biomedical system. My research indicated that these limitations were not necessarily related to actual practices; rather they have more to do with the constraints of the environment in which the IM practices. For complications they cannot solve, logistics such as lack of transport, especially during the rainy season, make it extremely difficult to obtain the help that they know is needed. Yet the Karamojong IMs were extremely experienced at recognising problems in pregnancy such as fetal malpresentation and expert at correcting this. I give as an example the situation described below, which happened on my way to a meeting with a group of Indigenous midwives, to which this elderly IM belonged. I passed her hut on the way and stopped to give her a lift, but:

*Swat came to say that she would not be able to come along until later. She became very excited and began waving her arms about and holding two fingers of each hand up. She made massaging gestures on her own abdomen and cradled her arms as if holding a baby. Anna, my translator, joined us at that point and began to interpret for me. Swat had been up all night delivering two sets of twins. One pair was born to her own daughter and the other to her daughter-in-law. One of the twins was transverse—a serious problem that would have stumped most professional midwives. Swat had reached in, pulled the baby’s feet down, swept its arms up so they would not get stuck, and successfully delivered the baby. Both mothers and all four babies, she assured us, were in a good condition. She was obviously very happy with the situation and had managed that potentially disastrous challenge without any fuss and with minimal light, going back and forth between two mud huts in the wee hours of the night.*

This occurrence not only demonstrated this Indigenous midwife’s skill but also her confidence in dealing with what to a Western practitioner would be an extremely problematic situation necessitating hospital transport. It was evident that an emergency of this magnitude was not unfamiliar to Swat, yet the situation did highlight the need for a referral mechanism that could be accessed rapidly. However, even if this could be organised (and eventually was), the logistical problems caused by the rainy season could not ignored, as some areas become isolated as the formerly dry riverbeds become unpassable raging torrents. This again makes it clear that the services of the IMs cannot be simply swept aside. The fact always remains that at times she is all the woman has for her survival if a complication occurs.

### The Gradual Erosion of the Traditional Midwife’s Power

The high regard in which the IMs of Karamoja have traditionally been held is being gradually eroded as a result of the maelstrom of changes in this society brought about by literacy and exposure to outside influences. In addition, contact with biomedical services by women of childbearing age has generated the belief in many that hospital birth is in itself necessarily better than birth in the village. An aspect of this is also the prestige associated with the ability to pay with money for services, rather than to pay "in kind” with goods, as is done with the TBAs. Their traditional power depends on the clear public recognition of their identities both as respected elder women and as midwives. Parallel to this perception are emergent factors that question the extent of their power—namely, the changing perceptions of the IMs by the women and the changes to which these midwives themselves are exposed. The impacts of education and exposure to Western ideas have created ambivalence in the women’s own understandings and attitudes toward the IMs and to the services provided by the biomedical system. This uncertainty is compounded by the reality that “choice” is never more than provisional due to the logistical realities of the area. It would seem that in this context of change, the power of the IM is definitely being modified and perhaps even diminishing, precisely because of the modernizing transitions going on in her society. However, the IM takes the opportunity, on occasion, to demonstrate her power, and to, so to speak, “go public.” This is what happened during the workshop held from 30 October to November 3, 1990:

*Day three of this event was used to give an opportunity for the Community Health Care Co-ordinator to discuss the programme for the area--e.g., vaccination, leprosy and tuberculosis control--with the traditional midwives. The co-ordinator, himself a Karamojong, had previously met with the TBAs and taken part in some of my meetings with them. He was therefore familiar with the other aspects of the programme. He adapted his discussion so that the focus was directed to the promotion of health and prevention of disease in pregnancy. He spent the morning talking with the TBAs about various vaccinations available to the female population, such as anti-tetanus and malaria prophylaxis.*
*This had been a lively session which ended in the sharing of refreshments. The programme was for the traditional midwives to spend an afternoon some 6 km away at a place where the community gathered for vaccination sessions. We ferried the group of 20 midwives by Land Rover to the vaccination venue and left them with the vaccination team to observe the session. When we went to collect these midwives at the end of the afternoon, they had not only learned how the vaccination schedule was organised but had, in addition, spontaneously examined all the pregnant women who attended. When we arrived, the atmosphere was very happy and like a celebration. What had initiated this activity was the recognition of the traditional midwives by the pregnant women in the area. The momentum grew as the afternoon progressed, and women who had been vaccinated passed the word on that “the Akimats were helping.”*

Thus, out of an educational activity for the IMs, there also developed greater confidence in the vaccination programme, due to the support and endorsement of the midwives. This constituted a powerful example of their credibility, power, and influence within the community. The vaccination team later reported that it was the best community response to vaccination they had ever had in that area. Subsequent discussions with the IMs revealed that they intended to encourage women to go forward with vaccination for themselves and their children.

The evidence suggests that this positive and supportive attitude of the IMs toward the health care programme sprang from the months spent beforehand in building up communication and relationships among them, the hospital, and Community Based Health Care services (CBHC). It is significant that the IMs, while exercising their own powers, also demonstrated their confidence in the CBHC activities. This factor was later influential when the TBAs’ programme was further developed and extended to neighboring areas of South Karamoja, and other IMs came to support the work of the CBHC. As I worked with groups of IMs in different regions, I always made a point of telling each group about the other. This often led to their choice to meet, get to know each other, share practices, and come to support the CBHC.

Such an outcome suggests that when a biomedical practice comes to be culturally perceived as valuable, it will be promoted by local people themselves. Yet the unfortunate reality is that time is not often budgeted for local education in development programmes and the full potential of the programme is therefore not realised.

### Biomedical Affirmation and the Development of Mutual Accommodation

It was recognised by the local medical authorities in Karamoja that most births were managed by a TBA. This was the main reason I was originally approached by these authorities and asked to help. The Partnership Paradigm I developed in conjunction with the IMs and the biomedical staff was in response to the IMs who had identified the situations in which they needed help. This happened during a process of regular discussions with the IMs and workshops attended both by them and by biomedical staff. Mutual respect grew out of the developing relationship between the biomedical practitioners and the Karamojong IMs, which, in keeping with Karamojong custom, took place within a dialogical context and the recognition that authoritative knowledge should not be the prerogative of one system alone. I acknowledged both the strengths and values of the traditional midwives’ model of care and those of the biomedical system, and initiated the dialogue between them, which led to the creation of a practical working partnership between the village midwives and the biomedical health services. This approach was key to my plan to develop a mutually beneficial scheme for the care and wellbeing of mothers and babies in the area. Patience was needed to allow for a change in consciousness by both parties and a willingness to cooperate with the change in perception that was needed.

The methods employed for achieving this partnership involved ongoing meetings with the TBAs, community health care workers, and hospital personnel. During this process, the TBAs made observation visits to the hospital and got to know the staff, and they in turn visited the community and attended some of the meetings with the TBAs. The process was interesting and enjoyable. I made arrangements for the TBAs to come to the hospital in pairs. On their arrival, they were courteously greeted by the medical Superintendent (the main obstetrician and surgeon) in his office. He welcomed them and expressed appreciation for their work. Then each TBA was linked with a hospital midwife, and they spent a morning together in the antenatal clinic. The exercise was a great success for them both. When I went to find them, they were talking excitedly about the morning’s work, which had obviously been enjoyable. As the clinic began, the TBAs simply took over examining the women as they arrived. Fortunately, the hospital midwife allowed this involvement and was in fact both surprised and deeply impressed by the skills of the TBAs. Her comment was “These old women really know, don’t they?” This comment clearly signalled the new unity between the two groups.

After 18 months of building links and understandings between the hospital personnel, community health care workers, and the TBAs, I organised a one-week workshop for the TBAs, hospital midwives and community staff, who acted as facilitators for the discussions and the role play sessions that were the basis of our methodology. There was a key discussion with a hospital doctor about serious indications for immediate referral of women to hospital, during which the IMs expressed their deep concerns about problems such as haemorrhage in pregnancy and after delivery, to which the doctor responded.

By the time we held this week-long workshop, the TBAs had begun to view themselves as part of a publicly identifiable group instead of just being known as individual TBAs in their own communities. Once this notion was internalized, they began to sense the potential power they might hold as a group. They demonstrated their new sense of group identity in various ways—the most colourful being that they sang loudly all through their journeys from and to their homes to our meeting place either on foot or when I was able to give them a lift in the Land Rover. Their singing and exuberance attracted public attention; their songs were always about their work and how I had brought them together. It was difficult to get an accurate translation of what they sang, but it went something like this:

*Sally Locham, Sally Locham (I had two Karamojong names:“Locham” and Moru.” I was told they meant “mountain” and “top dog”!)*
*She brought us together,*
*We are the birth attendants*
*Yes, Oh ahh, Oh aah…..*

This refrain was repeated over and over and taken up by all the women as they met up on the road. Sometimes they varied the words to include something about the discussion we had just had and how they were looking forward to the coming workshop. Their pride in being part of a group overrode their fear of being professionally upstaged by one another. The group was of mixed ability and experience; this factor emerged during the workshop, as will be seen below in my description of the role playing. Like professionals anywhere, they have secrets and various “tricks of the trade,” which they were previously not willing to share.

Their pride in being publicly recognised elicited a need for concrete evidence of their status. This was expressed in a request for t-shirts bearing the logo (in English) “Traditional Birth Attendant.” I was able to procure these, and they were ceremoniously distributed with great joy during the closing ceremony of the week-long workshop. In reciprocity—another strong value of the Karamojong—they presented me with a goat skin to wear on ceremonial occasions. Again, this demonstrated an attitude of mutuality.

The dialogical approach used throughout encouraged participation by everyone. In this way, the week-long workshop became an enriching and learning experience for all the participants, biomedical and Indigenous alike. One of the main aims of the week was for the IMs to demonstrate, by discussion and role play, their practices and the cultural ceremonies they carried out during pregnancy, labor and the postnatal period. These demonstrations then were used as a starting point for discussions and linked up with midwifery practices and the variety of problems the TBAs encounter.

Roleplay throughout the workshop was the most effective way of exchanging ideas and demonstrating practices. It was interesting and enjoyable, and gave a sense of reality and continuity to the ceremonials, which were acted out by the IMs. At each phase of the proceedings, they worked in groups and identified their criteria for seeking medical help and referral to hospital. The hospital staff went through the same process, demonstrating hospital procedures and identifying criteria for transfer. This process showed the two cultures in stark contrast. On the one hand, it showed birth firmly embedded in a village context using only the artefacts of everyday life; on the other hand, the clinical approach epitomized the biomedical culture. After the roleplay sessions and group work, there was general feedback to the whole group. The criteria for referring women to the hospital were agreed upon after a lengthy discussion. This customarily slow dialogic process gave equal merit to the views of both groups, so that the decisions made represented the perceived needs of each.

When practice issues were discussed, the hospital staff shared their own clinical procedures for the management of serious problems and described the treatments they employed. There was no attempt made to persuade the TBAs to change their own methods, but rather to offer some alternative approaches that they might find more effective than the ones they used. For most situations, as the biomedical staff learned, the TBAs’ treatments were already effective and based on sound physiological principles. For example, for the treatment of post-partum haemorrhage, they understood well that it was vital for the woman’s uterus to contract if bleeding was to be controlled. As previously noted, they achieved this by massaging the uterus, and in cases of extreme necessity by manual removal of the placenta. They also claimed that the herbs they gave to drink had the same effect as the biomedical drugs used in the hospital. It was not possible to prove this but, on the final day of the workshop, a TBA named Elizabeth, who was also a respected herbalist, brought along a selection of roots and herbs and laid them out for us to see. In exchange, I displayed various tablets such as analgesics, antibiotics, and stimulants for making the uterus contract. We discussed the relative merits of each. Some of the herbs were used for the same purposes as the drugs I showed for such complications as postpartum infection. The other TBAs said that they were familiar with some of the herbs, such as Tamarin, and used them in their practice, but others were new to them. They also spoke confidently about the beneficial effects of some of their own herbal remedies for postpartum haemorrhage and fever.

During the discussion on the relative merits of tablets and herbs, since most of the group had received effective biomedical treatment for illnesses such as chest infections and malaria, they vouched for the efficacy of some of the drugs. Again, the intention of our knowledge exchange was to share with the TBAs what could be beneficially incorporated into their practices, to raise awareness about situations where early referral might prevent serious problems such as obstructed labor, and to educate the biomedical staff as to what the TBAs already knew and understood. The partnership we conjointly developed between the IMs and the biomedical practitioners sought not to change the biosocial nature of the IMs’ model, but rather to support it. Reciprocation within this partnership was demonstrated when the hospital recognised the feasibility of allowing the IMs to accompany their clients in the hospital to provide continuity of care, and adopting some of the Karamojong cultural practices during hospital birth—for example regarding disposal of the placenta, which the IMs often chose to take with them for burial near the woman’s home, as was the custom. This reciprocity also influenced the position in which the woman gave birth. Instead of requiring Karamojong women to be prone, biomedical staff assisted the IM in helping the woman to remain upright.

And, once they understood the reasoning, the IMs were happy to abandon their traditional cord-cutting practices in favor of cutting the cord with a fresh razor blade and tying it with a ligature. Even more important than these concessions was the agreement that the TBAs were welcome to come to the hospital with the woman and to stay with her throughout the labor, including being present at caesareans.

The TBAs’ recognition of the value of biomedical help for complications they were unable to manage indicated that changes had occurred in how they viewed complications and how to deal with them. Such changes in their perceptions were further indicated by the requests made by the TBAs: the first was for help with transporting laboring women from the villages to the hospital in the case of a serious emergency; the second was for personal identity cards stating that a request for urgent help was being made by a TBA. They asked that when this card was presented to hospital personnel, it would evoke a quick response and transport would be provided by the hospital without delay. These two requests were accepted by the hospital authorities and the details later worked out and implemented. The TBAs felt it necessary to have this particular arrangement rather than to simply send a verbal request for help, because the demands on the hospital are numerous, resources of both transport and personnel are limited, and some requests are not responded to because they are often “false alarms.” Thus the staff have to be selective and try to discern how to respond when asked for help. The TBAs knew this and could foresee the potential delay in response, and so felt that both special arrangements and trust in their judgments were needed to avoid delay.

## The Official Liaison Between the Hospital and the Traditional Birth Attendants

The October 1990 workshop launched the first phase of official public liaison between the TBAs and the hospital. At the end of the weeks’ discussions, the occasion was celebrated by a feast, dancing, singing, speeches, and the public acknowledgement by the hospital staff of the TBAs as valued colleagues. Each TBA was presented with an identity card containing her own photograph and a collection of other cards that could be sent by the TBA via a messenger to the hospital when requesting emergency help. These items were very well received by the TBAs. There were mutual expressions of commitment and friendship between the hospital medical and midwifery staff and the TBAs. The exchange of speeches and celebration is not viewed as a social nicety by the Karamojong, but as a serious commitment and bond of friendship that carries with it mutual responsibilities. Once begun in this way, co-operative links became established and were maintained by our monthly meetings with the TBAs in the villages.

One of the deep values inherent in the Karamojong model of midwifery is that the IM is truly a “*with-woman”:* a companion, friend, comforter and trustworthy attendant throughout the whole process of childbearing. This close attention and concern happen in the normal course of events; women see each other every day and it is quite common for the IMs to carry out daily examinations of a pregnant woman. This familiarity means that by the time labor begins, a strong rapport has been built up between the midwife and the mother, whereas the continuity of care inherent in the biosocial model of Karamoja and of other Indigenous societies has largely been sacrificed in the biomedical model in favor of the intensive use of technology. The use of technology, nonetheless, is not to be deprecated but rather evaluated within the overall context of care.

Diversification can be a strength and is to be encouraged to meet the differing situations and circumstances of birth. If viewed in this way, the potential for building relationships between models can lead to interactivity and mutual support—what Jordan (1993) called “mutual accommodation”—between differing systems. This is clearly preferable to the dominance and replacement by one at the expense of the other, and is attainable when the will is there to strive for it.

Let it also be clearly noted here that the biomedical model of maternity care as found in Karamoja and in most low resource countries cannot be compared to the sophisticated, efficient systems found in high-resource countries. Very often, because of poor training of personnel, lack of motivation and incentive, poor funding, faulty technology, scarcity of resources, troubled situations and other disadvantages, these biomedical centres can be very detrimental, even dangerous, in their low-resource context. Hence, one must not think that the biomedical model in a low-resource area is the panacea for all ills. The success of the Partnership Paradigm was due in large part to the excellence of the biomedical centre (a missionary hospital) to which one could confidently refer for help, as well as to the openness and respect extended to the traditional midwives by the hospital authorities and their staff.

Karamoja presents a combination of factors that in many ways mitigate against the notion of partnership. The fact that it was achieved despite these factors supports my assertion that the Partership Paradigm is not only feasible but also *portable—*applicable anywhere in the world where traditional midwives practice and there is a will among biomedical practitioners to establish dialogue and develop mutual respect over time. This creative approach brings maternity care into line with the recommendations of Alma Ata principals of situating care in and near the people. As has been clearly demonstrated, *women everywhere want care in their communities* (Davis-Floyd et al., 2009).

## Conclusion

Sally Graham and Robbie Davis-Floyd

Given the realities of life in Karamoja and in many other parts of the Global South, it can be—and has been—disastrous for functional and effective (in most cases) traditional systems to be phased out and replaced by biomedicine in the interests of modernization and the global push to facility birth. Rather than being phased out, traditional midwifery systems should be “phased in” and the TBAs clearly affirmed—that is, both systems ought to merge their strengths and values to be appropriate in the evolving circumstances of low resource countries. Birth for many traditional or Indigenous societies, including the Karamojong, is a social event accompanied by rituals and celebrations. To wrench it from this setting and contract it out to biomedicine will and has destroyed its wider community context, and with it those cultural expressions that are, or were, an inherent part of that culture. The technocratic biomedical model is not appropriate for people but rather for machines; in fact, as Robbie has long shown (Davis-Floyd, 2001; Davis-Floyd, 2003a), that model treats the birthing body as if it *were* a machine. The model of care applied to traditional societies must be culturally appropriate and safe. There must be an underpinning of anthropologically appropriate principles so that the inherent values of the culture are not sacrificed in the biomedicalization of birth.

The development of the Partnership Paradigm, which Sally, her biomedical colleagues in Karamoja, and the IMs fostered for many years, makes possible and practical both an enhancement and inclusion of all the viable IM skills, whilst at the same time respecting both the culture and the value of biomedicine when needed. The principle of partnership is there to facilitate further development of IMs’ knowledge and practice without destroying their autonomy or their cultural relevance. The process involves mutual enlightenment and the most effective use of resources. A similar model was developed by Australian midwife and researcher Lesley Barclay (2009) and her team in Samoa that dramatically reduced maternal mortality there, once the “bush midwives” and the nurse-midwives in the hospitals reached the same sort of partnership and mutual accommodation we are recommending. Another such model has been developed by an NGO and is working well in Guatemala (Daviss et al., 2021). Yet in most other low resource countries, TBAs continue to be sidelined.

The partnership paradigm that Sally helped to develop as a result of dialogue obviously benefits both systems and, over the ensuing years, has shown itself to in fact be the way forward for both biomedical practitioners and TBAs. We argue that the Partnership Paradigm is appropriate for *any* situation in which a biosocial model of care is operational alongside the biomedical one. Its development is straightforward, requiring only motivation, perseverance and willingness on the parts of both biomedical staff and TBAs. Sally was able to facilitate the generation of such conditions in Karamoja, just as Lesley Barclay and her team were able to do in Samoa and an NGO was able to do in Guatemala. It often only takes one or a very few people to strike the spark and set the process in motion, and then, again, it takes commitment, patience, and time to see it through.

### The Price of Eliminating Traditional Birth Attendants

Eliminating TBAs inevitably brings backlash. To replace the traditional midwives of Karamoja and many other countries, it would be necessary to also replace the rainy season, when the roads are impassable and the rural areas impenetrable. It must be taken into account that there are times every year in countries with climate extremes or with hard-to-reach rural villages when the TBA is required to take full responsibility for women in labor. This is not going to change, and, due to the Climate Crisis, may even intensify in the foreseeable future, however optimistic one is about facility birth. The demise of the TBA in such rural areas has often left the house empty of any maternity care providers. In places where TBAs have already been phased out of practice and biomedical facilities are both inaccessible and ineffective, the effects have been devastating: because no skilled birth attendants (SBAs) will practice in such communities, women are left completely without care. Robbie vividly recalls a Midwifery Today conference held in 2007 in Costa Rica, during which groups of traditional midwives from Honduras, Nicaragua, and other Latin American countries, who had been banned from practising, came to the microphone to tell tragic stories of labouring women walking for many hours down mountains and across rivers trying to get to a road where they could catch a bus to get to a hospital, and often giving birth in a field or dying on the way. And should they reach the hospital, they would receive racist and discriminatory treatment, often provided in a language they did not understand. Clearly, prohibiting local midwives in rural areas is not a way to reduce maternal mortality.

## Postscript 2021

After Sally’s initial research and work in Karamoja (1988–1991), she visited yearly for the next 10 years and was able to help a new local trainer facilitate further development of the groups. During these years, the IMs worked closely with the community-based health care program and the hospital. The number of identified IMs reached 120 practising in six outlying districts, meeting regularly, attending workshops and reporting their work to the trainer. Their contributions helped to reduce the huge gap that had previously existed between the biosocial and the biomedical models of care. The strategy of working in partnership led to appropriate referral of pregnant women to the hospital for assessment, successes in child immunization and antenatal care, improvements in hospital deliveries, and the lowering of maternal and infant mortality.

This Partnership Paradigm prevailed in Karamoja until, in 2010, the Ugandan government issued a policy that banned all TBA trainings and also banned TBAs from attending births. Nevertheless, the Karamojong TBAs continued to practice “under the radar” and their good relationships with the biomedical staff also continued. However, training was discontinued and there are no longer any TBAs officially connected to the hospital. The free ambulance services for pregnant women and neonates continue.

As previously noted, it was the ban on TBA training that prevented Sally from publishing this work much sooner, as she was afraid that government policy would destroy what had been accomplished. And in fact, despite the efficacy of Sally’s initiative, this has happened to some extent, yet all has not been lost. Some TBAs have been absorbed as members of the Village Health teams. Some of their roles in this capacity include family visits to give health education, to encourage pregnant women to go for antenatal care, to refer those with danger signs for a hospital delivery, and to visit mothers and babies for postnatal care. Some also work with NGOs as leaders educating other mothers on health issues and linking them to facilities for services. With a focus on neonatal and child health, they also treat children below five who are suffering from malaria, pneumonia and diarrhoea at home and refer severe cases to health facilities.

Yet despite the legal ban on the TBAs conducting deliveries and the government policy to ensure that all women are attended at birth by a qualified midwife, in Karamoja, the environmental and logistical challenges still exist and the TBAs continue to attend births, especially during the rainy season. It is regrettable that their extensive skills can no longer be “officially” harnessed alongside those of the professional midwives working in the hospital. The Partnership Paradigm worked well for 20 years, and still exists, only now it operates with extreme caution due to the misguided policies of the Ugandan government, which are the same as those of many other low resource countries. We close by again recommending full and official integration of TBAs in countries where they continue to exist, and the implementation of the Partnership Paradigm.

## Ethical Approval

Graham’s fieldwork was conducted from 1988 to 1991 and during multiple subsequent visits with the approval of the biomedical staff, community health workers, and the Karamojong people she worked with and studied, long before the days of IRBs. Written approval for the publication of this article has been given by the executive team at St Kiztio Hospital, Karamoja, where much of this work was carried out.

## Data Availability

The datasets presented in this article are not readily available because this dataset is not available to the public. Requests to access the datasets should be directed to davis-floyd@outloook.com.
